# Supplementary material for: Burdens and Resources of Staff of a Specialized Ward for Neuropalliative Care: A Cross-Sectional Survey
Source: Brain Sci. 2022 Dec 10;12(12):1697. doi: 10.3390/brainsci12121697 (PMC9776069; doi:10.3390/brainsci12121697)
Supplement: Supplementary file 1 [file brainsci-12-01697-s001.zip › brainsci-1975537-supplementary.pdf]

Supplementary Material:

Supplementary Material Table S1: Modifications to the original questionnaire (Ateş et al. 2020, see Appendix, section 7.4, page 71-76)

Original questionnaire	***Modifications***
paper based	digital, web-based
Wie stark fühlen Sie sich durch folgende Faktoren belastet?, page 71 *(To what extent do you feel burdened by the following factors?)*	
Widersprüchliche Behandlungspläne im Versorgungsnetz (Question 16)	Widersprüchliche Behandlungspläne im Versorgungsnetz (auf Station)<br>*(we clarified by "auf Station" that the question addresses the work at the ward)*
Wie viele Sterbefälle im Rahmen Ihrer Arbeit halten Sie persönlich aus, ohne dass es zu einer Überlastung führt?, page 71 *(How many deaths can you personally endure without becoming overwhelmed?)*	
Für alle Einrichtungen/Dienste außer ambulanter Hospizdienst ___ Patient*innen pro Woche<br>Für ambulante Hospizdienste ___ Patient*innen pro Monat	___ Patient*innen pro Woche<br><br>*(the differentiation of bearable deaths between inpatient and outpatient care had a risk of being misunderstood, therefore question was simplified to only "patient per week" at the ward)*
Wenn Sie an Patient*innen oder Zugehörige denken, welche Faktoren belasten Sie?, page 72 *(When you think of patients or their relatives, what factors make you feel burdened?)*	
Zerrissenheit / kein Zusammenhalt in der Familie (Question 7)	Zerrissenheit / kein Zusammenhalt in der (betroffenen) Familie<br>*(we clarified by "betroffenen" that the question addresses the patient´s family)*
Begleitung bei bestimmten Erkrankungen (Question 13)	Die Begleitung von Patient*innen mit folgenden Erkrankungen empfinde ich als (leicht, stark oder sehr stark) belastend. *(we specifed the question)*
Haben sich Ihrer Einschätzung nach die Arbeitsbedingungen an Ihrem Arbeitsplatz/in Ihrem Ehrenamtlichen-Dienst seit Ihrer Anstellung/Ihrem Eintritt verbessert oder verschlechtert?, page 71 *(In your opinion, have the working conditions at your place of work/in your voluntary service improved or deteriorated since you were hired/began?)*	Haben sich Ihrer Einschätzung nach die Arbeitsbedingungen an Ihrem aktuellen Arbeitsplatz geändert?<br><br>*(we simplified the question "Do you think the working conditions at your current place of work have changed?")*
Demographic data, page 75	
- Geschlecht *(sex)*<br>- Wie alt sind Sie? ______Alter in Jahren *(How old are you? ______Age in years)*<br>- Welches Herkunftsland haben Sie? *(What is your country of origin?)*<br>- Welches Herkunftsland haben Ihre Eltern? *(What country of origin are your parents from?)*	Staff surveys in our hospital require Human Resources approval. The approval was granted under the condition that information on sex, age and country of origin must not be collected.
In welchem Umfang sind Sie derzeitig im hier befragten Dienst beschäftigt? page 76 *(To what extent are you currently employed in the service interviewed here?)*	In welchem Umfang sind Sie derzeitig beschäftigt?

Supplementary Material Table S2: List of all questioned potential burdens.

**Questioned potential burdens:**	
patient- and relative-specific:	*organizational, institutional, personal, collegial:*
similarities with me or people close to me	too many patients
particularly close relationships with patients	too few patients
particularly difficult relationships with patients	accumulation of deaths in a certain period of time
too high demands of others on my care	no time to address wishes and problems of those affected
other cultural background	too short periods of accompaniment (<3 days) until death
conflicts in the (affected) families	unexpected death of patients
socially isolated patients	Memories of deceased persons in their own environment
particularly close relationships with patients' relatives	not being able to accompany patients until they pass away
particularly difficult relationships with patients' relatives	feelings of guilt
communication difficulties	burden caused by suffering of colleagues
too high own demands on my care	burden caused by own suffering
particularly difficult relationships with relatives	patients with good prognosis rejecting suggested therapies
low/lack of family support	too little staff (incl. unfilled positions)
	too little time for rituals
	high documentation effort
	conflicting treatment plans within the care network
	unsuccessful treatment of symptoms
	start or continuation of therapies in patients with poor prognosis

Supplementary Material Table S3: Stress symptoms shown by the team when confronted with death. Importance of symptoms was dichotomized in "not at all/slightly" and "(very) much". *not significant after p-value adjustment to p=0.004 using Bonferroni-correction for multiple testing.

	**NPC-ward**			**Neuro-ward**			**Fisher's exact test**
	Not at all/ slightly	(very) much	Response rate	Not at all/ slightly	(very) much	Response rate	
Speechlessness	85%	15%	100%	77%	23%	94%	n.s.
Irritability	79%	21%	95%	67%	33%	94%	n.s.
Increase in arguments	88%	12%	85%	70%	30%	94%	n.s.
Accusations	80%	20%	100%	71%	29%	97%	n.s.
Self-withdrawal	89%	11%	95%	82%	18%	88%	n.s.
Excessive talkativeness	68%	32%	95%	68%	32%	97%	n.s.
Rejection of usual rituals	83%	17%	90%	83%	17%	91%	n.s.

No involvment with other or new patients	94%	6%	85%	87%	13%	97%	n.s.
Cynicism	84%	16%	95%	67%	33%	94%	n.s.
Work-to-rule	65%	35%	85%	64%	36%	88%	n.s.
No acceptance of offers of support	87%	13%	80%	73%	27%	81%	n.s.
Refusal of new admissions to ward	100%	0%	80%	93%	7%	94%	n.s.
Increased interprofessional tensions	95%	5%	95%	68%	32%	97%	p=0.023*
Crying	94%	6%	100%	87%	13%	97%	n.s.

Supplementary Material Table S4: Occurrence of physical and mental distress symptoms within the last four weeks on the NPC-ward and the Neuro-ward dichotomized in "never/occasionally" and "(very) often". No statistically significant differences (p<0.05) were found.

	**NPC-ward**		**Neuro-ward**		**Fisher's exact test**
	Never/ occasionally	(very) often	Never/ occasionally	(very) often	
Rushed or under time pressure	50%	50%	31%	69%	n.s.
Depressed	65%	35%	65%	35%	n.s.
Calm and balanced	65%	35%	66%	34%	n.s.
A lot of energy	70%	30%	63%	37%	n.s.
Physical pain	65%	35%	65%	35%	n.s.
Completely exhausted after work	40%	60%	44%	57%	n.s.
Sleep disturbances	60%	40%	56%	44%	n.s.
